# Low-Dose Exposure and Immunogenicity of Transgenic Maize Expressing the *Escherichia coli* Heat-Labile Toxin B Subunit

**DOI:** 10.1289/ehp.9687

**Published:** 2006-12-19

**Authors:** April J. Beyer, Kan Wang, Amber N. Umble, Jeffrey D. Wolt, Joan E. Cunnick

**Affiliations:** 1 Interdepartmental Microbiology, Iowa State University, Ames, Iowa, USA; 2 Plant Transformation Facility, Department of Agronomy, Iowa State University, Ames, Iowa, USA; 3 Biosafety Institute for Genetically Modified Agricultural Products, Iowa State University, Ames, Iowa, USA; 4 Department of Animal Sciences, Iowa State University, Ames, Iowa, USA

**Keywords:** adulterated food, genetically modified organism, heat-labile toxin, inadvertent exposure, oral vaccine, plant vaccine, transgenic maize, transgenic plant

## Abstract

**Background:**

Transgenic maize, which produces the nontoxic B subunit of the *Escherichia coli* heat-labile toxin (LT-B) in seed, has proven to be an effective oral immunogen in mice. Currently, there is considerable concern over accidental consumption of transgenic maize expressing LT-B by humans and domestic animals. We have yet to define nonimmunogenic levels of transgenic LT-B when ingested.

**Objectives:**

Our goal in this study was to determine the highest dose of LT-B orally administered in mice that does not result in a measurable immune response. We defined an immune response as specific serum or mucosal IgG or IgA significantly greater than background after three feedings (0.0002–20 μg) or a priming response induced by the intermittent feeding.

**Methods:**

We fed transgenic maize pellets on days 0, 7, 21, and 49 and collected serum and fecal samples weekly. Serum was analyzed for LT-B–specific IgG and IgA, and feces was analyzed for LT-B–specific IgA.

**Results:**

We observed a dose-dependent anti-LT-B antibody response with high specific antibody concentrations in groups fed high doses (0.2, 2, 20 μg) of LT-B maize. Mice fed 0.02 μg LT-B demonstrated immune priming in 62.5% of the animals. Mice that were fed ≤ 0.002 μg LT-B showed no increase in specific antibody nor did they demonstrate immune priming, indicating that 0.002 μg LT-B was the highest nonimmunogenic dose tested.

**Conclusion:**

Our results demonstrate that LT-B derived from transgenic maize is immunogenic at nanogram levels when orally administered to mice.

Many pathogens enter the body via mucosal surfaces. To prevent infection via this route, a protective secretory IgA response is required. Traditional parenteral vaccines primarily induce IgM and IgG responses, whereas mucosal vaccination, for example via the nasal or oral route, is able to elicit both an IgG and a secretory IgA response. Recently, expression of vaccine antigens in plants has been explored as an approach to facilitate oral vaccination (Kong et al. 2001; Ma et al. 2003; Tacket et al. 2000; Webster et al. 2002).

Plant-based oral vaccines have several advantages over traditional parenteral vaccines. Their production is more economical and their delivery is safer, as needles and animal products are not used (Lauterslager et al. 2001; Streatfield et al. 2001). Plant cells also provide protection to the antigen during long-term storage, transport at ambient temperature, and as it passes through the harsh environment of the stomach (Chikwamba et al. 2003). Most important, plants do not carry pathogens that are harmful to humans and animals (Streatfield et al. 2001).

Our work focuses on transgenic maize expressing the B subunit of the *Escherichia coli* heat-labile toxin (LT-B). Previous studies have shown that this maize is both immunogenic, eliciting the production of secretory IgA upon oral administration to mice, and protective against challenge with the holotoxin (LT) (Chikwamba et al. 2002). LT-B is expressed within the starch granules of the maize seed as demonstrated by Chikwamba et al. (2003). Because of this natural encapsulation, maize-expressed LT-B is protected against harsh environmental conditions including high temperatures and enzyme degradation, while purified recombinant LT-B is not (Chikwamba et al. 2003). In addition, the natural encapsulation in maize starch granules may provide better protection and stability than other plant-produced vaccines such as potato, tomato, or tobacco. This increased stability should lead to improved immunogenicity.

The enhanced stability of maize-expressed LT-B is highly desirable for oral vaccine production, storage, and effectiveness; however, LT-B and its stability are also a concern in regard to environmental contamination and/or accidental consumption by humans and domestic animals. Safety issues remain that must be addressed. The use of transgenic maize to produce a vaccine component opens the possibility for low-dose antigen exposure of workers involved in its production or of consumers should the product inadvertently occur in foods (Wolt et al. 2006). Low-dose exposures have implications for product efficacy, which we address in this article. Furthermore, as immunogenicity is highly sensitive, evidence of immunogenic effects at low doses helps to set the lower bound for subsequent considerations of dose-dependent allergenic potential. Thus, we must determine the minimum dose for which no immune stimulation occurs upon accidental consumption.

The work we present here focuses on determining the immunogenicity of maize transgenic for LT-B in an intermittent feeding schedule and identifying a maximum nonim-munostimulatory dose. We hypothesized that some low levels of LT-B could stimulate non-detectable levels of specific antibody but result in immune priming—an ability to induce the production of memory B cells that can later respond to the same antigen and produce specific IgG or IgA. We defined immune priming as a 4-fold increase of antibody (IgG or IgA) over background after a 20-μg LT-B booster exposure (Tacket 2005; Tacket et al. 1998, 2004). Because of the high degree of specificity and sensitivity of the immune response to the highly immunogenic LT-B, doses of maize-derived LT-B that do not prime or stimulate the immune response should be considered an environmentally safe threshold.

## Materials and Methods

### Preparation of maize pellets

Production of maize transgenic for LT-B (seeds from 4th-generation transgenic plants) and preparation of the maize seed pellets were carried out as described previously (Chikwamba et al. 2002). The pellets were formed by combining appropriate amounts of ground transgenic maize seed with ground nontransgenic (nt) maize seed to ensure that each pellet was a consistent size and contained the amount of LT-B indicated for each group. For the second study, we prepared the lowest doses (0.0002–0.02 μg LT-B) by mixing the ground transgenic maize seed with ground nt maize seed 1:300 to make a lower LT-B level transgenic mix. The total weight of maize seed in each pellet was 0.811 g for the first study and 0.937 g for the second study. The ground maize seed for each pellet was mixed with 600–700 μL of phosphate-buffered saline (PBS) [1.9 mM NaH_2_PO_4_, 8.1 mM Na_2_HPO_4_, 0.15 M NaCl (pH 7.3)], formed into a pellet, and air-dried overnight. Ground nt maize seed was used to make pellets of similar weight to feed to the negative control groups.

To ensure that LT-B content in the maize remained constant during pellet formation, one extra pellet was formed per dose per feeding for analysis of LT-B content using a ganglioside-dependent ELISA. Three to four pellets of each type were frozen until ELISA analysis of LT-B content (four samples from each pellet). We detected no significant differences between the intended amount and the amount measured with ELISA for all pellets except the 20-μg pellets. The 20-μg pellets had higher amounts of LT-B than intended. These amounts ranged from 26.5–40.8 μg LT-B per pellet instead of 20 μg (*p* < 0.0001; data not shown). Despite the range of measured LT-B in the 20-μg pellets, we will continue to refer to this dose as the nominal 20-μg dose and animals given that dose as the 20-μg group.

### Oral immunization of mice

We obtained 4- to 6-week-old female BALB/c mice from Harlan Sprague Dawley (Indianapolis, IN, USA) and allowed them a 2-week acclimation period with a 12-hr reversed light/dark cycle before beginning the experiment with lights on at 2100 hr. The mice were housed four per cage in the Iowa State University (ISU) animal facility with food and water *ad libitum*. Animals were treated humanely, and all procedures were approved by the ISU Institutional Animal Care and Use Committee.

Before the maize seed pellets were fed to the mice, the mice were fasted overnight (during the light phase) with water *ad libitum*. While eating the pellets, mice were housed individually with water *ad libitum* and were allowed to eat the pellet for 4 hr or until the pellet was finished, typically a maximum of 6–8 hr for the slowest mice. Upon completion of the pellet, the mice were returned to group cages with mouse chow and water *ad libitum*.

In the first study, we divided mice into six groups of four mice each. The experimental groups were fed 0.02, 0.2, 2, or 20 μg LT-B per pellet per feeding. The two control groups were fed nt maize pellets. The maize pellets were fed on days 0, 7, and 21. To test for immune priming, on day 49 all mice were fed a 20-μg LT-B pellet, including one group previously fed nt maize (nt + 20). In addition, one nt group was fed nt maize pellets on day 49.

In the second study, we used the same experimental design except that some mice were fed lower doses of LT-B. The groups consisted of mice fed 0.0002, 0.002, 0.02, and 20 μg LT-B per pellet per feeding and two groups fed nt maize. To test for immune priming, on day 49 the mice were all fed 20 μg LT-B, except one nt group. Thus, internal repeats of the nt, nt + 20, 0.02-, and the 20-μg groups were included for a total of eight mice receiving each of these treatments over two experiments.

### Sample collection and preparation

We collected serum and fecal pellets before the initial dosing and weekly throughout both studies to detect LT-B–specific serum IgG and IgA concentrations and fecal IgA concentrations. Blood was collected via the saphenous vein, using heparinized capillary tubes and centrifuged with serum separator gel (Vacutainer Plus plastic serum tube; Becton Dickinson, Franklin Lakes, NJ, USA) at 6,000 × *g* in a bench top microcentrifuge (Jouan M14.11; Thermo Electron Corp., Waltham, MA, USA) for 10 min. The serum was collected in micro-centrifuge tubes and stored at –20°C until analyzed for LT-B–specific IgG and IgA by ELISA as described below.

Fecal pellets were collected from each mouse, frozen, lyophilized, and stored at –20°C. We extracted lyophilized pellets by adding extraction buffer [0.05% NaN_3_, 10 μg/mL leupeptin (Sigma, St. Louis, MO, USA), 0.25 mM PefablocSC ( Sigma) in PBS] at 10 μL/mg of lyophilized feces, vortexing, and extracting overnight at 4°C. Before analysis, the samples were placed on a shaker (Tekmar VXR-S10; Janke & Kunkel, Staufen, Germany) at 1,000 rpm for 90 min, then centrifuged at 6,000 × *g* for 10 min with serum separator gel. The liquid extract was collected and analyzed by ELISA for LT-B–specific IgA as described below.

### Euthanization

Mice were euthanized on day 57 for the first study; for the second, half the mice were euthanized on day 55 and the other half on day 56. For both studies, mice were euthanized with CO_2_.

### Lung lavage

After mice were euthanized we performed lung lavages by exposing the trachea, inserting a TomCat catheter (Kendall Sovereign, Tyco Healthcare, Mansfield, MA, USA), and flushing the lungs twice with 0.8 mL sterile PBS. The samples were centrifuged at 6,000 × *g* for 10 min to remove cells and stored at –20°C until analysis for LT-B–specific IgA by ELISA as described below.

### ELISAs

We performed ELISAs for LT-Band LT-B–specific antibodies as previously described (Chikwamba et al. 2002; Karaman et al. 2006) unless otherwise noted. All mouse samples, which were analyzed individually, and reagents were added 50 μL/well, and between each step wells were washed 4 times with 100 μL/well PBST [0.05% Tween 20 (polyoxyethylene sorbitan monolaurate; Sigma) in PBS].

#### Detection of LT-B–specific IgG and IgA

Measurements of LT-B–specific IgG (serum samples) and IgA (serum, fecal, and lung lavage samples) were carried out as described previously (Karaman et al. 2006) with minor changes. Briefly, all fecal extracts were measured within 24 hr of extraction. Sample concentrations were determined by comparing to a standard curve consisting of wells coated with either purified mouse IgG (MOPC 21, Sigma) with a range of 1.37–1000 ng/mL or IgA (Bethyl Laboratories, Montgomery, TX, USA) with a range of 0.46–400 ng/mL. Samples were diluted appropriately to fall within the linear range of the curve. End point readings were taken at 405 nm using the EL 340 microplate reader (Bio-Tek Instruments, Inc., Winooski, VT, USA) and data collected using KC Junior software (version 1.17, Bio-Tek Instruments, Inc.) using a four-parameter fit standard curve. Samples reading below the standard curve were reported as one-half the value of the lowest detectable standard to permit log transformation of the data for statistical analysis.

#### Detection of LT-B in maize

We extracted LT-B from ground maize or crushed maize pellets equivalent to those used for feeding using a sodium phosphate extraction buffer [sodium phosphate buffer (pH 6.6) 25 mM; sodium chloride 100 mM; EDTA, 1 mM; Triton X-100, 0.1%] with protease inhibitors (leupeptin, 10 μg/mL; PefablocSC, 0.25 mM) at 10 μL/mg maize. Samples were extracted with buffer shaking at 1,000 rpm at 37°C for 2 hr. The extract was collected by centrifuging at 6,000 × *g* for 15 min in a bench top microcentrifuge.

We determined LT-B content of maize extract using a ganglioside-dependent ELISA as described previously (Chikwamba et al. 2002) with minor changes. LT-B was detected by incubating with rabbit anti-LT-B antibody (diluted 1:10,000; Immunology Consultants Laboratory, Inc., Newberg, OR, USA), then incubating with biotin-conjugated goat anti-rabbit IgG (diluted 1:5000; Sigma). The secondary antibodies were detected using streptavidin-horse radish peroxidase (diluted 1:1000; Becton Dickinson), then incubating with ABTS (3-ethylbenzthiazoline-6-sulfonic acid; Sigma) substrate buffer [0.1 M citric acid, 0.55 mM ABTS (pH 4.25)]. Values were determined by comparing to a standard curve of purified bacterial LT-B (provided by J. Clements).

### Statistical analysis

We analyzed the data with general analysis of variance, using LT-B dose and sample day as variables in the model. Because of unequal variance between the groups, the antibody data were log transformed before analysis (Kirk 1982). Each sample day that was significantly different (*p* < 0.05) from the prefeed date was further analyzed by between group contrasts for that day. Analysis was conducted using the statistical software Statistix (version 8; Analytical Software, Tallahassee, FL, USA). We used non-log-transformed data for graphs. Immune responses were divided into two types and defined as follows: An antibody response is demonstrated by antibody concentrations significantly higher (*p* < 0.05) than those of the nt-fed group, and immune priming is demonstrated by antibody concentrations 4-fold higher than those of the nt-fed group 5–6 days after a 20-μg LT-B dose (Tacket 2005; Tacket et al. 1998, 2004).

## Results

### LT-B–induced dose-dependent response with immune priming

In our first experiment we tested the immunogenicity of various doses of LT-B from transgenic maize. To identify a nonimmunostimulatory dose, we tested 10-fold differences of LT-B doses (ranging from 0.02 to 20 μg per feeding) using an intermittent feeding regimen previously shown to induce a robust antibody response in 28 days with pellets fed on days 0, 7, and 21 (Chikwamba et al. 2002).

#### Serum IgG

**Figure 1:**
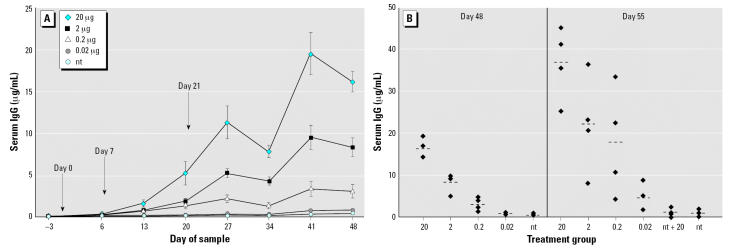
Study 1 serum IgG concentrations in response to three intermittent feedings of LT-B (*A*) or a booster dose of 20 μg LT-B given on day 49 (*B*). (*A*) LT-B feeding days are indicated by the arrows. Antibody concentrations are presented as mean ± SE. (*B*) Group means are represented as bars (- - -). For both *A* and *B*, *n* = 4 mice per group, except for the nt group, which had *n* = 8 on days –3 to 48.

Intermittent feeding of LT-B induced a dose-dependent serum IgG response (Figure 1A). Mice fed 20 μg LT-B had low serum IgG levels (0.318 ± 0.061 μg/mL) but these levels were significantly higher than those of mice fed nt maize (0.169 ± 0.018 μg/mL) by day 6 (*p* = 0.0035) and were significantly higher than all other groups on days 13–48 (*p* < 0.05). Mice fed 2 and 0.2 μg LT-B had significantly higher concentrations of IgG by day 13 compared with the nt group (*p* < 0.0001 for both groups on days 13–48). The concentrations of specific IgG in the 0.02-μg group were low, but demonstrated a significant increase on day 27 (0.298 ± 0.039 μg/mL) compared with the nt group (0.159 ± 0.018 μg/mL) (*p* = 0.0038), with significance continuing through day 48 (*p* ≤ 0.0089).

The peak antibody concentration for all groups was reached on day 55, 6 days after the 20-μg booster as seen in Figure 1B. After the booster, LT-B–specific IgG levels were significantly increased for mice in the 20-, 2-, 0.2-, and 0.02-μg groups compared with the nt group (*p* ≤ 0.0065). All mice in the 20-, 2-, and 0.2-μg groups demonstrated antibody levels at least 4 times higher than nt mice on day 55. In the 0.02-μg group, two of four (50%) mice had antibody levels 4 times those of the nt control group, which is indicative of immune priming. There was no statistical difference between either of the nt groups for serum IgG throughout the study, even after feeding one group 20μg LT-B on day 49 (*p* = 0.7085).

#### Serum IgA

**Figure 2:**
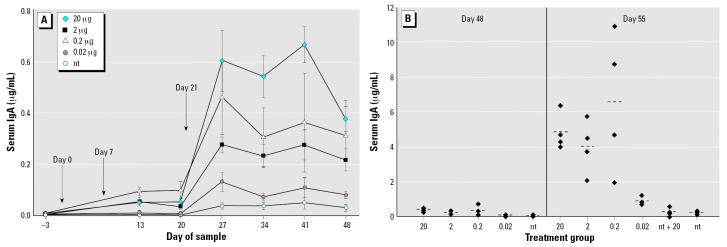
Study 1 serum IgA concentrations in response to three intermittent feedings of LT-B (*A*) or a booster dose of 20 μg LT-B given on day 49 (*B*). (*A*) LT-B feeding days are indicated by the arrows. Antibody concentrations are presented as mean ± SE. (*B*) Group means are represented as bars (- - -). For both *A* and *B*, *n* = 4 mice per group, except for the nt group, which had *n* = 8 on days –3 to 48.

As with serum IgG, feeding LT-B generally induced a dose-dependent response in serum IgA (Figure 2A). Significantly increased antibody levels compared with those in the nt group were observed in the 20-, 2-, and 0.2-μg LT-B groups beginning on day 13 and continuing to the end of the study (*p* ≤ 0.0071) (Figure 2A,B). The 0.2-μg group demonstrated higher but not significantly different levels of antibody compared with the 2-and 20-μg groups on days 13 and 20. Additionally, the antibody serum IgA levels of 0.2-μg group remained higher than those of the 2-μg group until the end of study. Although we are not sure why the lower dose resulted in higher antibody levels, we are certain that it was not a technical error, as the same serum samples demonstrated a dose-dependent IgG response. We hypothesized that perhaps a lower dose, such as 0.2 μg LT-B, is optimal for stimulating an early IgA response Also, the high average value was not just due to one outlier in the 0.2-μg group, as on day 13, three of four mice in the 0.2-μg group had higher antibody levels than the mice in the 2-μg group for serum IgA.

Because of the short half-life of IgA, obtaining a high level of IgA antibody significantly different from that in the nt group was not always observed with consecutive sample dates. This was demonstrated by the 0.02-μg group, which was marginally different from the nt group (*p* = 0.0785) on day 13 and significantly different on days 27, 34, 48, and 55 (*p* ≤ 0.0351). On day 55, three of four (75%) mice in the 0.02-μg LT-B group appeared to have responded to the 20-μg booster dose with LT-B–specific IgA levels 4 times higher than those of the nt control group (Figure 2B), suggesting that these three mice were immunologically primed. There was no significant difference between the nt and nt +20 groups throughout the study (*p* = 0.9481 on day 55) and none of the mice (zero of four) in the nt + 20 group had antibody levels 4 times higher than those of the nt group on day 55. Because of a lack of samples, no data from day 6 of the study were obtained.

#### Fecal IgA

**Figure 3:**
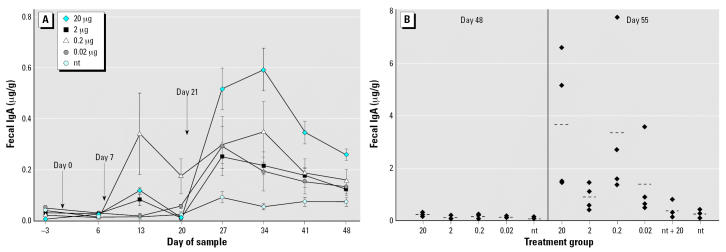
Study 1 fecal IgA concentrations in response to three intermittent feedings of LT-B (*A*) or a booster dose of 20 μg LT-B given on day 49 (*B*). (*A*) LT-B feeding days are indicated by the arrows. Antibody concentrations are presented as mean ± SE. (*B*) Group means are represented by bars (- - -). For both *A* and *B*, *n* = 4 mice per group, except for the nt group, which had *n* = 8 on days –3 to 48.

Mucosal IgA was measured in extracts of lyophilized fecal material and expressed as micrograms per gram of fecal material as shown in Figure 3. Mice fed 20 μg LT-B had significantly higher levels of IgA in fecal material than those of the nt group on day 13 (*p* < 0.0001) and again on day 27 throughout the rest of the study (*p* ≤ 0.0019). The 2-μg group had significantly higher levels than those of the nt group on days 13, 27, 34, 41, and 55 (*p* ≤ 0.0371). Similar to the serum IgA results, mice fed 0.2 μg LT-B had levels of fecal IgA that were significantly higher than those of the nt group by day 13 (*p* < 0.0001) and throughout the remaining sample dates (*p* ≤ 0.0384).

Fecal IgA levels were statistically higher in the 0.2 μg group than those in the 2 μg LT-B group on days 13, 20, and 55 (*p* < 0.05). Again, this was not because of an outlier in the group, as on all those dates at least three of four mice in the 0.2-μg group had higher antibody levels than those of the mice in the 2-μg group.

Fecal antibody concentrations of mice fed 0.02 μg LT-B reached statistical significance on days 20, 27, 34, and 55 (*p* ≤ 0.0163) with antibody levels only marginally higher than the those of the nt group on days 41 and 48 (*p* = 0.0515 and 0.0712, respectively). On day 55, 1 week after the 20-μg booster, one mouse in the 0.02-μg group had 4 times the antibody concentration of that in the nt group (Figure 3B), suggesting that immune priming had occurred in one of four mice. The nt and nt + 20 groups were not statistically different throughout the study (*p* = 0.7278 on day 55).

### Confirmation of 0.02-μg threshold for immune priming

During the second intermittent feeding study, antibody responses to low doses of LT-B administered orally in transgenic maize were measured. Because our first study demonstrated a significant increase in antibody concentrations and immune priming in some mice fed 0.02 μg of LT-B, we chose to repeat this dose and test two doses, each 10-fold lower in order to identify a nonimmunogenic dose. We also included a 20-μg LT-B dose as a positive control. Thus, two groups were repeated from the first experiment and two groups were new. As in the first experiment, we looked for production of serum and fecal antibody as well as immune priming.

#### Serum IgG

**Figure 4:**
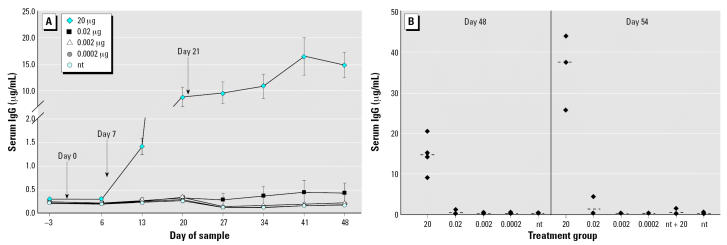
Study 2 serum IgG concentrations in response to three intermittent feedings of LT-B (*A*) or a booster dose of 20 μg LT-B given on day 49 (*B*). (*A*) LT-B feeding days are indicated by the arrows. Antibody concentrations are presented as mean ± SE. (*B*) Group means are represented by bars (- - -). For both *A* and *B*, *n* = 4 mice per group, except for the nt group, which had *n* = 8 on days –3 to 48.

As seen in Figure 4A, mice fed 20 μg LT-B had significantly elevated levels of serum IgG (0.296 ± 0.035 μg/mL) by day 6 (*p* = 0.0046) compared with the nt group (0.201 ± 0.017 μg/mL) and had significantly higher levels throughout the study (*p* < 0.0001 for all remaining sample dates), reaching peak levels on day 54 that were similar to those observed in the first study (Figure 4B vs. Figure 1B). The 0.02-μg group had marginally higher levels than those of the nt group on days 27, 41, and 48 (*p* = 0.0535, 0.0594 and 0.0514, respectively) and significantly higher levels on days 34 and 54 (*p* = 0.0335 and 0.0257, respectively). No other groups had IgG concentrations significantly different from those of the nt group (Figure 4A,B). On day 54 for the 0.02-μg group, one of four (25%) animals demonstrated an antibody level 4 times that of the nt group (4.2 μg/mL vs. 0.2 μg/mL), suggesting that immune priming had occurred in that animal (Figure 4B). Despite that one animal in the nt + 20 group demonstrated elevated antibody concentrations after the 20-μg LT-B booster, the increase may be the beginning of primary response because the level of antibody (1.4 μg/mL) is far below that of the primed mouse in the 0.02-μg group (4.2 μg/mL). Additionally, the nt + 20 group was not statistically different from the nt group.

#### Serum IgA

**Figure 5:**
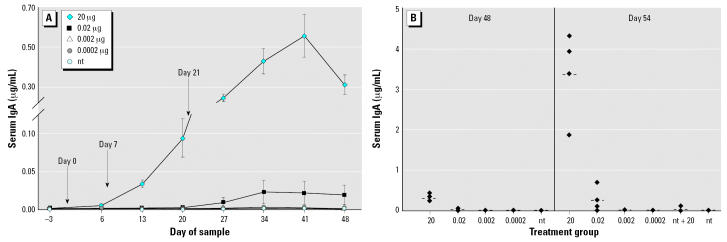
Study 2 serum IgA concentrations in response to three intermittent feedings of LT-B (*A*) or a booster dose of 20 μg LT-B given on day 49 (*B*). (A) LT-B feeding days are indicated by the arrows. Antibody concentrations are presented as mean ± SE. (*B*) Group means are represented by bars (- - -). For both *A* and (*B*, *n* = 4 mice per group, except for the nt group, which had *n* = 8 on days –3 to 48.

The serum IgA concentrations of the 20-μg LT-B group (0.5721 μg/mL) were significantly higher than those of the nt group (0.0607 μg/mL) by day 6 (*p* = 0.0088 on day 6 and *p* < 0.0001 for all remaining sample dates), as seen in Figure 5A. The antibody concentrations of the 0.02-μg group were marginally different from those of the nt group on day 27 (*p* = 0.0548) and significantly different on day 34 and all subsequent days (*p* ≤ 0.0294). No other group had antibody concentrations significantly different from those of the nt group throughout the study. Once again the 0.02-μg LT-B group had two of four (50%) mice that appeared to be primed (Figure 5B). To calculate the level of antibody 4 times higher than those of the nt group for this data set, we used the data from the nt + 20 group, as all mice in the nt group had no detectable antibody on day 54. No other group (0.0002 or 0.002 μg) had responders by this standard.

#### Fecal IgA

**Figure 6:**
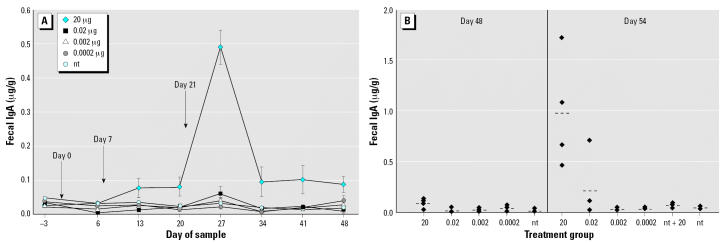
Study #2 fecal IgA concentrations in response to three intermittent feedings of LT-B (*A*) or a booster dose of 20 μg of LT-B given on day 49 (*B*). (*A*) LT-B feeding days are indicated by the arrows. Antibody concentrations are presented as mean ± SE. (*B*) Group means are represented by bars (- - -). For both *A* and *B*, *n* = 4 mice per group, except for the nt group, which had *n* = 8 on days –3 to 48.

The 20 μg LT-B group had antibody concentrations marginally higher than those of the nt group by day 13 (*p* = 0.0574) and significantly higher by day 20 (*p* = 0.0046) and throughout the remainder of the study (*p* ≤ 0.0202), as seen in Figure 6A. The 0.02-μg LT-B group did not have a significant increase in antibody concentrations compared with those of the nt group; however, after the 20-μg booster, one of four (25%) mice responded with 4 times the antibody concentrations of those of the nt group, suggesting immune priming (Figure 6B). No animals fed lower doses of LT-B responded by either measure.

#### IgA from lung lavages

**Figure 7:**
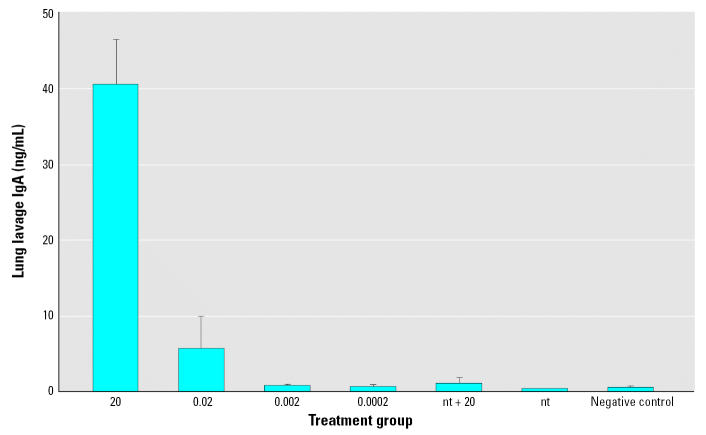
Study 2 lung lavage IgA concentrations on day 54 in response to a booster dose of 20 μg LT-B given on day 49. Antibody concentrations are presented as mean ± SE with *n* = 4 mice per group. The groups are labeled according to the amount of transgenic maize fed on days 0, 7, and 21 in micrograms. The group nt + 20 is the nt group fed 20 μg on day 49. The nt group is the nt group fed all nt maize. The negative control group was never handled, fasted, or fed maize.

Mucosal IgA was measured in lung lavage fluid of euthanized mice (Figure 7). The 20- and 0.02-μg LT-B groups had significantly elevated levels of IgA compared with those of the nt group and the negative control group, which was never fasted, handled, or fed maize pellets (*p* < 0.0001 and *p* = 0.0029, respectively).

### Frequency of immune priming with 0.02 μg LT-B

Although the fecal IgA measurements in the second study for the 0.02-μg group were not significantly higher than those of the nt group, this dose resulted in significant levels of antibody in all other measures for both studies at least at one sample day before the 20-μg booster as well as by day 54 or 55. Additionally, approximately half the mice in both studies were primed, based upon antibody levels at least 4-fold higher than those of the nt group. One mouse in each study demonstrated priming using all three measures (serum IgG, serum IgA, and fecal IgA), whereas all other mice responded in only one or two of the measures (Table 1). The rates of immune priming varied between different measures (serum IgG vs. serum IgA vs. fecal IgA), indicating that some are more sensitive than others. We found serum IgA to be the most sensitive, which is likely because oral exposure to an antigen primarily elicits an IgA response as opposed to IgG. Also, fecal IgA is difficult to measure accurately because of the high rate of antibody breakdown in the feces. Using our most sensitive measure, serum IgA, we observed that the overall rate of immune priming in mice fed 0.02 μg LT-B intermittently is 62.5%.

#### Effect of maize consumption on mouse weight

Upon euthanization, the weight of each mouse was recorded. For both studies, we found no significant difference between mice fed transgenic maize compared with mice fed nt maize (*p* ≥ 0.14). In addition, for the second study, no significant difference was detected between mice that were handled, fasted, and fed transgenic or nt maize compared with mice that were never handled, fasted, or fed maize (*p* ≥ 0.11), thereby demonstrating that the nt and transgenic maize at the doses administered had no toxic effects on the mice (data not shown).

## Discussion

In this study we addressed the immunologic effects in mice of accidental consumption of low doses of maize transgenic for LT-B. The doses fed to the mice included 20 μg LT-B, a dose protective in vaccine studies, as well as five additional doses, with each 10-fold lower than the previous one (2, 0.2, 0.02, 0.002, and 0.0002 μg LT-B). The 20-μg dose was included in both experiments as a positive control. Chikwamba et al. (2002) and Mason et al. (1998) previously demonstrated that 10–50 μg LT-B fed 3 times intermittently to mice results in a protective level of antibody. Here we demonstrate that the 20-μg functional dose also results in a robust memory response that elicited 2-fold increases in serum IgG and 10-fold increases in serum and fecal IgA. Serum IgA represents a monomeric nonse-creted form of IgA. Although the biological function of serum IgA is unclear, differences in the concentration of serum IgA between animals correlates with differences in mucosally secreted IgA.

We have extended previous studies (Chikwamba et al. 2003; Karaman et al. 2006) by testing doses of 0.0002, 0.002, 0.02, 0.2, 2, and 20 μg LT-B for serum IgG and IgA as well as fecal IgA and we have observed a dose-dependent antibody response. Only mice that were fed ≥ 0.02 μg LT-B elicited significantly elevated levels of antibody. It should be noted that although the antibody response in the 0.02 μg group was low, it was significantly higher than that of the nt group in six of seven measures. The biological relevance of the low antibody concentration is underscored by the fact that approximately half the mice in the 0.02-μg group demonstrated a 4-fold increase in antibody when fed a 20-μg dose. No lower dose resulted in an antibody response or immune priming. From this study, we found that serum IgA was the most sensitive measure for detecting mice that had been immunologically primed. In addition, we identified 0.002 μg as the highest nonimmunostimulatory dose, one that resulted in neither an antibody response nor immune priming when fed intermittently.

Although we considered 0.02 μg LT-B a dose that results in immune priming, not all the mice in these groups resulted in a priming response. Some animals had fair antibody responses while others appeared to have no response at all on days 54 or 55. One explanation for this is that there is some variability even between genetically inbred BALB/c mice that becomes obvious when feeding a dose that is borderline for inducing a response. Alternatively, the amount of LT-B in the maize fed to each mouse may not have been uniform. Our group has observed that the finer the transgenic maize kernels were ground, the more LT-B could be extracted and detected in ELISA (Chikwamba et al. 2002). Although the extra pellets that we prepared and assayed were all very close to containing 0.02 μg LT-B (0.0226, 0.0184, and 0.0195 μg), perfect homogenous mixes are not practical using ground maize and it is not inconceivable that one or more of the experimental pellets may have been more variable in LT-B content. This is more of a problem with borderline doses than with the high, immunogenic doses used as a functional vaccine.

Although there are many advantages to producing LT-B in maize, there are also concerns associated with genetically modified organisms (Schmidt 2005). Because of the high immunogenicity of LT-B, even at low levels, intermittent accidental consumption of maize transgenic for LT-B could result in an antibody response, immune priming, or both depending on the dose ingested. Whether immune priming has a positive or negative effect on future vaccine administrations has yet to be determined. We can hypothesize that it would boost the immune response to a future vaccination meant to protect animals or humans from the LT holotoxin; however, if LT-B were to be used as an adjuvant in a vaccine against a heterologous antigen, the response to that vaccine may be altered. Additionally, further research is needed to ensure that LT-B administered orally will not promote the development of tolerance to vaccines or allergies to co-administered food proteins.

This study provides data that are the first of their kind to begin assessing the consequences of accidental consumption of LT-B in maize. Risk assessment for noncancerous, nontoxic transgenic plants is evolving and includes the use of uncertainty factors to extrapolate from animals to humans (Kodell and Gaylor 1999). A recent risk assessment by Wolt et al. (2006) of human exposure to LT-B in transgenic maize indicates that a dose 200-fold lower than a functional dose in humans is small enough to consider that maize unadulterated from a toxicologic perspective. However, in our mouse study, a dose 1,000-fold lower than the functional 20-μg LT-B dose, 0.02 μg LT-B was immunogenic and caused immune priming in approximately half the mice. The highest nonimmunostimulatory dose tested was 10,000-fold lower than the functional 20-μg LT-B dose. Using this information, we can estimate a safe, nonimmunogenic dose for humans. Tacket et al. (2004) fed maize transgenic for LT-B to humans. The functional dose in this study was 1.1 mg LT-B per 70-kg adult. A dose 10,000-fold lower than this functional dose is equivalent to 0.11 μg LT-B, which is our best estimate for a nonimmunogenic dose in humans.

Consuming low immunostimulatory doses of LT-B does not result in adverse toxic effects, as indicated by no changes in animal body weight or overall health; however, the low dose may be enough to alter immune responses. An immune response to LT-B may not be harmful on its own, but if accidental consumption were to alter the way in which a person or animal would later respond to an oral vaccine containing LT-B in transgenic maize, it could render the vaccine less useful. For example, daily exposure to LT-B may induce oral tolerance. If accidental consumption of transgenic LT-B maize were to occur daily (as opposed to the intermittent feeding schedule used in this study), this could lead to vaccine inefficiency as well as a reduced ability of the immune system to eliminate an ETEC infection.

The data presented here apply to the intermittent feeding schedule used. We understand that mice exposed to the same doses of LT-B at differing intervals may have different immune responses. Further studies are needed to compare intermittent and continuous feeding regimens for transgenic maize.

## Figures and Tables

**Table 1:** Frequency [no of mice (%)] of immune priming in mice fed 0.02 μg LT-B 3 times intermittently.

	Experiment		
Sample	1	2	Total
Serum IgG	2/4^a^ (50)^b^	1/4 (25)	3/8 (37.5)
Serum IgA	3/4 (75)	2/4 (50)	5/8 (62.5)
Fecal IgA	1/4 (25)	1/4 (25)	2/8 (25)

a Number of primed mice/total number of mice tested.

b Percentage of primed mice of total mice tested.
